# Odontological Society of Pennsylvania

**Published:** 1895-02

**Authors:** 

**Affiliations:** Odontological Society of Pennsylvania


### ODONTOLOGICAL SOCIETY OF PENNSYLVANIA.

THE regular monthly meeting of the Society was held November 10, 1894.

After the close of the routine business of the Society, the President, Dr. Pierce, stated that Dr. Henry Leffmann would read a paper on "Hydrogen Dioxide as furnished to Dentists."

(For Dr. Leffmann's paper, see page 78.)

DISCUSSION.

*The President.*—You spoke of acidity and non-acidity. Could you give us any idea whether the acidity of Marchand's peroxide is sufficient to have any deleterious effect?

*Dr. Leffmann.*—The acidity in Marchand's peroxide amounted to about eight in the test made to-day against about four, which is the minimum of the Oakland. The acidity of the pyrozone is about two for the same volume. Fifty cubic centimetres should not require more than five cubic centimetres of decinormal sodium peroxide to neutralize the acid present. Pyrozone shows about two, Oakland four, and Marchand's usually runs up to more than six or seven. To-day the sample, which was rather strong, ran to eight. If we were to dilute it to ten volumes it would reduce the acidity.

Marchand's article is undoubtedly being improved, and I think the later samples examined show a considerable decrease in the acidity, while the strength is greater.

A sample of Merck's, tested last February, was extremely acid. Two samples were obtained from different houses, and were found to have very little active oxygen (a little over one volume), but both very acid.

A sample of English hydrogen dioxide which I examined costs one dollar for a four-ounce bottle, and contains only about six volumes of active oxygen.

How imperfect must the treatment be that depends upon such inferior articles!

Dr. Deane produced a bottle of hydrogen dioxide, and said,—

"Since hearing this paper I would say I have here a bottle left by one of Marchand's men, and I used it in the ordinary way, and brought upon myself a great deal of trouble. I washed an abscessed bicuspid in the usual manner, and the patient not only lost the root, but the results were quite serious for ten days."

Dr. Leffmann asked if there were any of the material left.

Dr. Deane answered that there was, and Dr. Leffmann said he would be glad to take it and make an analysis of it.

*Dr. Leffmann.*—Dr. Thomas S. K. Morton received a sample of the Marchand article, left at his house by an agent, and it stood in a small side closet for some weeks. One of the members of his family became sick with diphtheria while at the sea-shore, and he was sent for. He thought he would take down, among other things, a bottle of hydrogen dioxide. He went to the closet and took up the sample unopened, and then, remembering the article I

had sent him, packing up a bottle of Oakland hydrogen dioxide, he took the train. About thirty minutes after he left, the people in the house heard a violent explosion. They went to the closet and found the bottle containing Marchand's dioxide torn into powder, and two or three other broken bottles lying on the floor. The decomposition had started, and the gas had gone on accumulating until the limit of pressure had been reached. Had that explosion occurred in his hands, it would probably have blinded him.

These accidents need not occur in properly made solutions. This sample may have been hydrozone. I find the hydrozone to contain a little less than thirty volumes.

I may say that so erroneous are the ideas on this point that when Dr. Wayne called upon one dealer, and at my suggestion asked for Oakland dioxide, he was told, "We don't keep it. Here is a brand that makes a big noise when you open the cork." That was a sign of decomposition. The best articles do not make a report upon lifting the cork.

Dr. Gaskill asked Dr. Deane if there was a fistula at the end of the root he referred to.

Dr. Deane answered that there was; that he was treating it from the outside through the fistula.

*Dr. Gaskill.*—Could you pass the fluid through the root?

*Dr. Deane.*—No; I was treating it from the outside.

*Dr. Gaskill.*—I used the sample, and used it very successfully, without any indication of trouble, and also found it valuable as a mouth-wash when there was inflammation of the gums. The patient used it three or four times daily in a diluted form, and after a few days the mouth showed a thoroughly healthy condition.

I forced it with the hypodermic through the root and fistula, and in twenty-four hours there was no sign of the opening to be seen on the gum.

Dr. Deane stated that he used it in two or three other cases, and found he was getting into trouble; that he had found the bottle always gave a slight explosion upon opening; and he thought if Dr. Leffmann, who had the bottle, would open it now an explosion would follow.

Dr. Gaskill stated that in his experience there was so much pop to it that the patient was startled, the cork being blown out onto the cabinet. He still used it, and with good results.

*Dr. Faught.*—I am sure we are all very much indebted to Dr. Leffmann for these accurate statements we have received in regard to hydrogen dioxide. I have used it in dental treatment, but my experience with the article has been largely in another direction. I began by using Marchand's solution, and always had a bottle of it in the house. I look upon it as a very good agent for application to the tonsils in their incipient inflammatory stages. I have one child who has a strong tendency to tonsillitis. Every winter he would lose his schooling and be in bed, while the younger children would be more or less affected. I reached the conclusion that a little care on my part would obviate his retirement from school to bed, so I began by keeping a bottle in the house in a cool place; yet on going to get the bottle I would very often find the cork blown out, even where it had been wired down. From the suggestions of Dr. Leffmann I substituted the Oakland Chemical Company's product, and I have had most excellent results. That article I have on hand constantly, and about once a week, or two or three times, just as opportunity occurs to me, I call the boys up in the morning and make an examination of their throats. If there is the slightest thready depositions, as we often see in cases of tonsillitis, I make an application with a swab that morning and the succeeding morning. I look upon it as prophylactic treatment to prevent the occurrence of diphtheria, and have always utilized hydrogen dioxide in this way to destroy germs upon the tonsils.

Dr. Luckie said he would like to ask Dr. Leffmann what the chemical results would be of mixing bichloride of mercury with pyrozone or hydrogen dioxide solutions, and if a good germicide could be obtained from such a solution.

*Dr. Leffmann.*—I have never made the experiment, but would be inclined to think you could make the mixture of corrosive sublimate with hydrogen dioxide, and that you would get a very efficient germicide that way. However, hydrogen dioxide is a peculiar body, and does not always mix well with certain substances that in theory ought to mix with it.

Dr. Pierce asked Dr. Leffmann if he understood him to say that the poorer solutions obtained were simply hydrochloric acid diluted.

*Dr. Leffmann.*—Yes, hydrochloric or sulphuric acid. Those noted to-night contained ten volumes, or three per cent. One-half volume would be one-twentieth of three per cent.,—a very small amount of material. The acid in that is an important ingredient. In all commercial hydrogen dioxide there is an acid. It does not keep if it is neutral; it does not keep in an entirely alkaline solution, but the acidity ought to be simply sufficient to preserve stability.

Joseph Head, M.D., D.D.S.,
*Editor Odontological Society of Pennsylvania.*

